# Supplementary material for: Membrane Attack Complex Mediates Retinal Pigment Epithelium Cell Death in Stargardt Macular Degeneration
Source: Cells. 2022 Nov 2;11(21):3462. doi: 10.3390/cells11213462 (PMC9655712; doi:10.3390/cells11213462)
Supplement: Supplementary file 1 [file cells-11-03462-s001.zip › cells-1986684-supplementary.pdf]

Supplementary Material

Title: Membrane Attack Complex Mediates Retinal Pigment Epithelium Cell Death in Stargardt Macular Degeneration

Ng & Kady et al.

CONTENT

TABLES:

Supplementary Table S1. Control and STGD1 patient variant information for additional SNPs correlated with AMD

Supplementary Table S2. qRT-PCR primers information

Supplementary Table S3. Antibodies information

FIGURES:

Supplementary Figure S1. Pluripotency, developmental, pigmentation markers, and ABCA4 expression and localization in human RPE cells cultured on 24-transwell inserts with growth medium without retinoids.

Supplementary Figure S2. Pulse-chase phagocytosis assay for human RPE cells.

Supplementary Figure S3. ABCA4-flippase activity in the RPE cells.

Supplementary Figure S4. Visual cycle proteins and activities in the RPE cells at three-months in culture.

Supplementary Figure S5. Complement C3a/C5a, CD59, and secreted proteins profile in human RPE cells.

Supplementary Figure S6. MAC (C5b-9) deposition accumulates in STGD1 RPE cells.

Supplementary Figure S7. Complement terminal complex destabilizes the RPE plasma membrane of the *Abca4*$^{-/-}$ mice.

Supplementary Table S1. Control and STGD1 patient variant information for additional SNPs correlated with AMD[a].

**Donor[a] Cell Line**	***CFH* 402**	***CFH* 62**	**ARMS2 A69S**	**C3 R102G**	**HTRA Promoter**	**SOD2**	**MBP**	**C8orf42 TDRP**
**Control**	YH	VV	N.D. [b]	RR	GG	VA	N.D. [b]	N.D.[b]
**STGD1**	YH	VI	N.D. [b]	RR	AA	VA	N.D.[b]	N.D.[b]

[a] Donor samples were genotyped for single nucleotide polymorphisms (SNPs) correlated with AMD (Complement factor H for SNPs 402 and 62; *ARMS2* - age-related maculopathy susceptibility 2, *HTRA1* - high temperature requirement factor A1, and *C3* – complement component 3) and for SNPs previously shown to be associated with geographic atrophy (near *SOD2* - Superoxide dismutase 2; near *MBP* - myelin basic protein; near *TDRP* - testis development related protein).

[b] N.D.: Not determined.

Supplementary Table S2. qRT-PCR primers information.

Gene	Accession		Primer Sequence (5’-3’)
Hum ABCA4	NM_000350.2	F	CCT GGA TGG GGA TGT AAA TG
		R	GGC ATA TGC TCT GTG CTT GA
Hum Oct 34	NM_002701	F	CTG TCT CCG TCA CCA CTC TG
		R	TGT GTT CCC AAT TCC TTC CTT AG
Hum Nanog	NM_024865.2	F	CCC TCC TCC CAT CCC TCA TAG
		R	TCG CTG ATT AGG CTC CAA CC
Hum Pax6	NM_001604.4	F	TTTGTCTTCCCTAGAAATCCTCAG
		R	TGATTGGACCGTGAACAGTAATAC
Hum OTX2	NM_0217728.2	F	ACCTTGAACTCCACCTCTGC
		R	GCTTCTCTTCTCTGACTCTCTTTG
Hum Tyr	NM_000372.4	F	GTG TAG CCT TCT TCC AAC TCA G
		R	GTT CCT CAT TAC CAA ATA GCA TCC
Hum Pmel17	NM_006928.3	F	GTT GAT GGC TGT GGT CCT TG
		R	CAG TGA CTG CTG CTA TGT GG
Hum PEDF	NM_002615.4	F	TATCACCTTAACCAGCCTTTCATC
		R	GGGTCCAGAATCTTGCCAATG
Hum GAPDH	NM_002046.3	F	AGC AAG AGC ACA AGA GGA AGA G
		R	GAG CAC AGG GTA CTT TAT TGA TGG
Hum PPIA	NM_021130.3	F	GTA CTT GGT GCT ACA GTC AGT C
		R	AGC TAG GGA GAG GCT CTA TAT G
Hum HMBS	NM_000190.3	F	TGC TAT CTG GGG AGT GAT TAC C
		R	GGC TGT TGC TTG GAC TTC TC
Hum GPI 3'	NM_000175.2	F	ATC TTC GTT CAG GGC ATC ATC
		R	CCA TCA AGC TCA GGC TCT ATT T

Supplementary Table S3. Antibodies information.

Primary Antibody	Company	Catalog #	Dilution (method)
ABCA4(2404)	Gifted from Dr. Hui Sun (1)	UCLA	1:100 (ICC)
ABCA4	Abcam	Ab72955	1:100 (ICC)
ABCA4	Everest Biotech Ltd	EB08615	1:25 (WES)
Alexa Fluor 488nm	Invitrogen	A11001	1:500 (ICC)
Alexa Fluor 568nm	Invitrogen	A11012	1:500 (ICC)
Alexa Fluor 647nm	Invitrogen	A21236	1:500 (ICC)
Alexa Fluor 647nm	Invitrogen	S21374	1:500 (ICC)
C3/C3b	MP Biomedicals	55463	1 µg/ml (WB)
C3a/C3a desArg	Abcam	Ab11873	1 µg/ml (WB)
C3aR	Abcam	Ab103629	1:100 (ICC)
C3aR (D-12)	Santa Cruz Biotechnology	SC-	1 µg/ml (WB)
C5aR/ CD88 (S5/1)	Santa Cruz Biotechnology	SC-53795	1 µg/ml (WB)
C5b-9	DAKO	M0777	1:50 (ICC)
C5b-9	Abcam	Ab55811	1:100 (ICC)
CD46	Biolegend	352403	1:100 (ICC)
CD46	Biolegend	315301	1 µg/ml (WB)
CFH	Quidel	A312	1 µg/ml (WB)
EEA1	BD Bioscience	610456	1:100 (ICC)
GAPDH	Abcam	Ab8245	1 µg/ml (WB)
4-HNE	ThermoFisher	MA52757	1:50 (ICC)
HRP anti-goat (kit)	Protein Simple	043-522-2	WES
HRP anti-rabbit (kit)	Protein Simple	042-206	WES
iC3b	Quidel	A209	1:100 (ICC)
IRDye® 680LT	LI-COR	926-	1:15,000 (WB)
IRDye® 680RD	LI-COR	926-	1:15,000 (WB)
IRDye® 800CW	LI-COR	926-	1:15,000 (WB)
IRDye® 800CW	LI-COR	926-	1:15,000 (WB)
IRDye® 800CW	LI-COR	926-	1:15,000 (WB)
IRBP	Santa Cruz Biotechnology	sc-25787	1 µg/ml (WB)
LRAT	Abcam	Ab73401	1:200 (ICC)
LRAT	Abcam	Ab137304	1 µg/ml (WB)
PDEF	Gifted from Dr. Joyce Tombran-Tink (2)	Penn. St. Univ.	1 µg/ml (WB)
PE-Duramycin	Molecular Targeting Technologies	D-1003	1 µ□M (ICC)
Peropsin	Gifted from Dr. Hui Sun (3)	UCLA	1:50 (ICC)
Phalloidin	Life Technology	T7471/A1	1:400 (ICC)
RAB5	Millipore Sigma	R-7904	1:75 (ICC)
RPE65	Millipore Sigma	MAB5428	1:150 (ICC)
RPE65	Gifted from Dr. Gabe Travis (4)	UCLA	1 µg/ml (WB)
Rhodopsin/1D4	Millipore Sigma	MAB5356	1:150 (ICC)
Vinculin	Abcam	Ab129002	1:5,000(WES)
ZO1	Invitrogen	40-2200	1:100 (ICC)

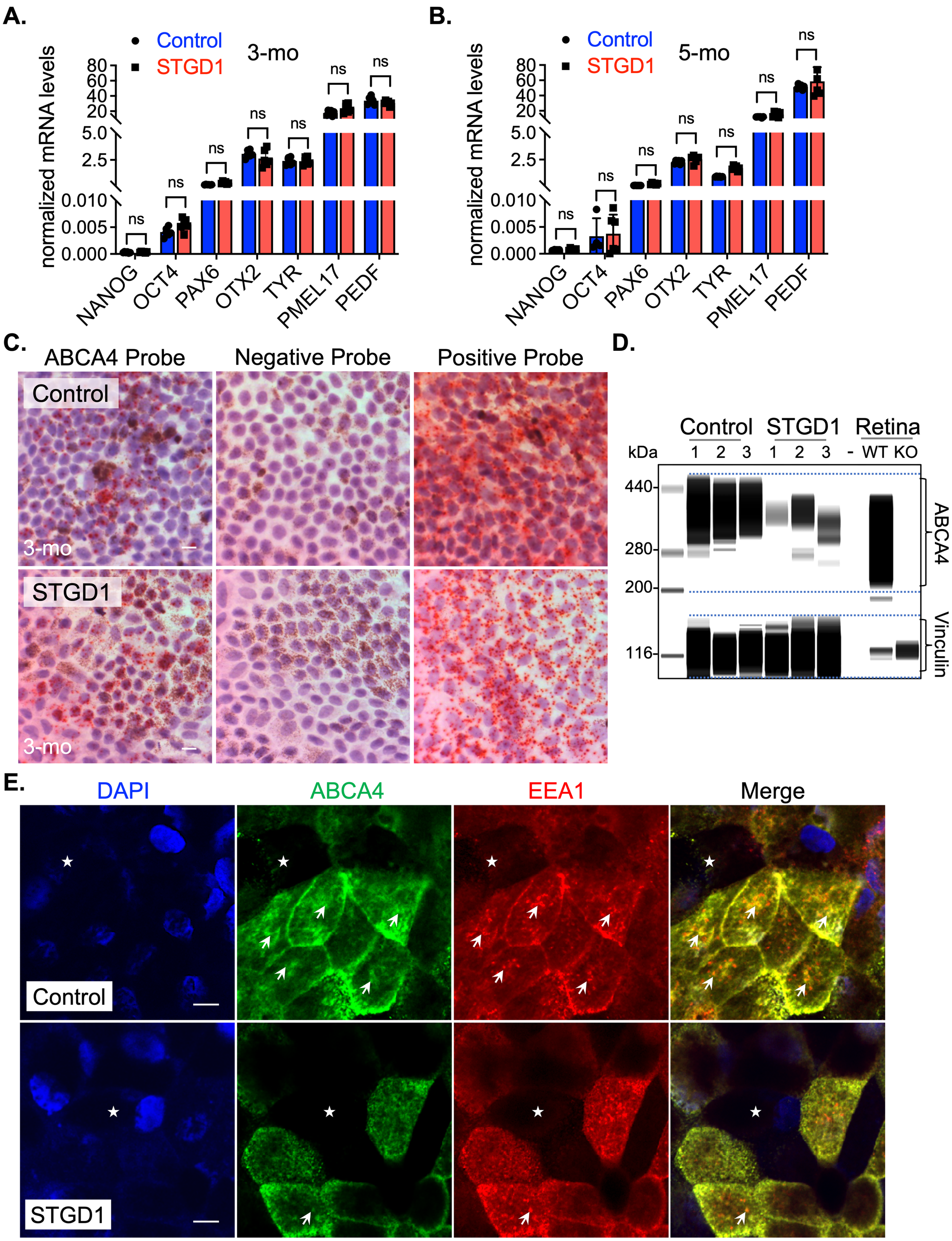

A.
Control
STGD1
3-mo
normalized mRNA levels
80
60
40
20
5.0
2.5
0.010
0.005
0.000
ns
NANOG
OCT4
PAX6
OTX2
TYR
PMEL17
PEDF
B.
Control
STGD1
5-mo
C.
ABCA4 Probe
Negative Probe
Positive Probe
Control
STGD1
3-mo
D.
Control
STGD1
Retina
kDa
1 2 3 1 2 3 - WT KO
440-
280-
200-
116-
ABCA4
Vinculin
E.
DAPI
ABCA4
EEA1
Merge
Control
STGD1

F.

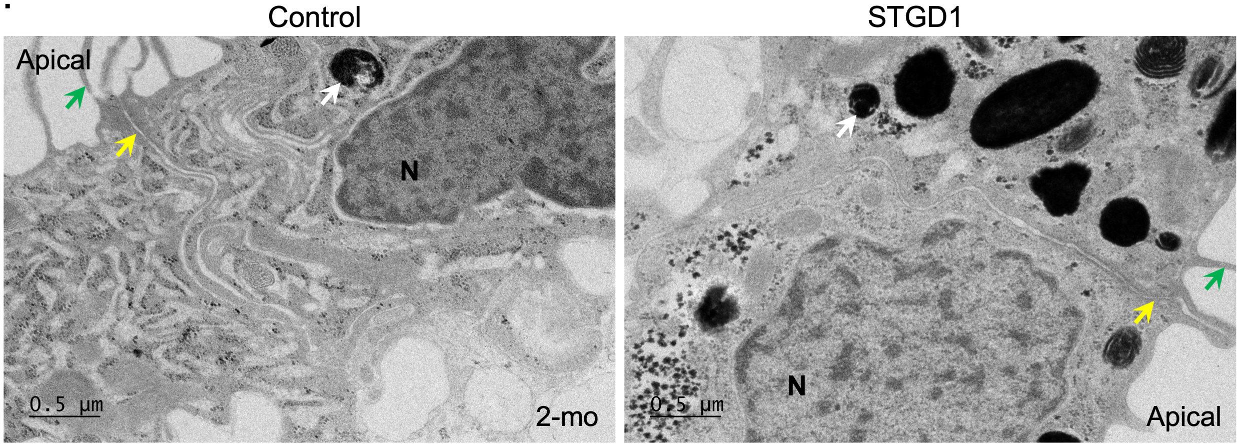

Supplementary Figure S1. Pluripotency, developmental, pigmentation markers, and ABCA4 expression and localization in human RPE cells cultured on 24-transwell inserts with growth medium without retinoids. Relative mRNA levels by qRT-PCR analysis of cDNA of the controls and STGD1 RPE cells at three-months (**A**, 3-mo) and five-months (**B**, 5-mo) respectively for various markers. OCT4, NANOG, PAX6, OTX2, TYR, PMEL17, PEDF mRNA levels were normalized to the geometric mean of four housekeeping genes: glyceraldehyde phosphate dehydrogenase (GAPDH), peptidylprolyl isomerase A, hydroxymethylbilane synthase, and glucose phosphate isomerase. Average data presented as mean ± SD; ns = not significant; n=6 filter/transwell per genotype for each time point. (**C**) In situ hybridization RNAscope chromogenic assay using three-months (3-mo) in culture RPE cells of control (top) and STGD1 (bottom) with target-specific probes for ABCA4 gene, bacterial dihydrodipicolinate reductase (Negative Probe), and polymerase II subunit A (Positive Probe). Similar red chromogenic reactivity is observed for both Control and STGD1 RPE cells for the ABCA4 and Positive Probe, while no red-staining is present with the Negative Probe; n=3 filter/transwell per genotype. (**D**) Representative digital image of WES capillary immuno-electrophoresis assay using three-months control and STGD1 RPE homogenates (20 μg) with an antibody against human ABCA4 protein; Vinculin was used as loading control and normalized data is shown in the main manuscript Fig. 1B; experiment was repeated twice; n=6 filter/transwell per genotype.

Numbers one to three on the digital image represent independent biological samples for each genotype. Wild-type (WT) and *Abca4*$^{-/-}$ (KO) retina homogenates were used as positive and negative control respectively in the WES assay. (**E**) Representative confocal images of fixed RPE cells of control (top row) and STGD1 (bottom row) grown for three-months in culture were stained with ABCA4 (green, Abcam antibody), EEA1 (red), and DAPI (blue for nuclei). Co-localization of ABCA4 and EEA1 is indicated by the white arrows and heavily melanin pigmented area by a white star. Co-localization is observed in STGD1 RPE cells also despite lower abundancy of mutated ABCA4 protein. Scale bar = 10 μm; n=3 filter/transwell per genotype; experiment was repeated twice. (**F**) Representative EM images of Control and STGD1 RPE cells taken at a higher magnification (6,000x); apical microvilli (green arrow), melanosomes (white arrow), junctional complexes (yellow arrow), and nucleus (N). Scale bar = 0.5 μm. Notable, both Control and STGD1 RPE cells are polarized displaying normal morphological features at two-months in culture with conditioned medium *without* retinoids.

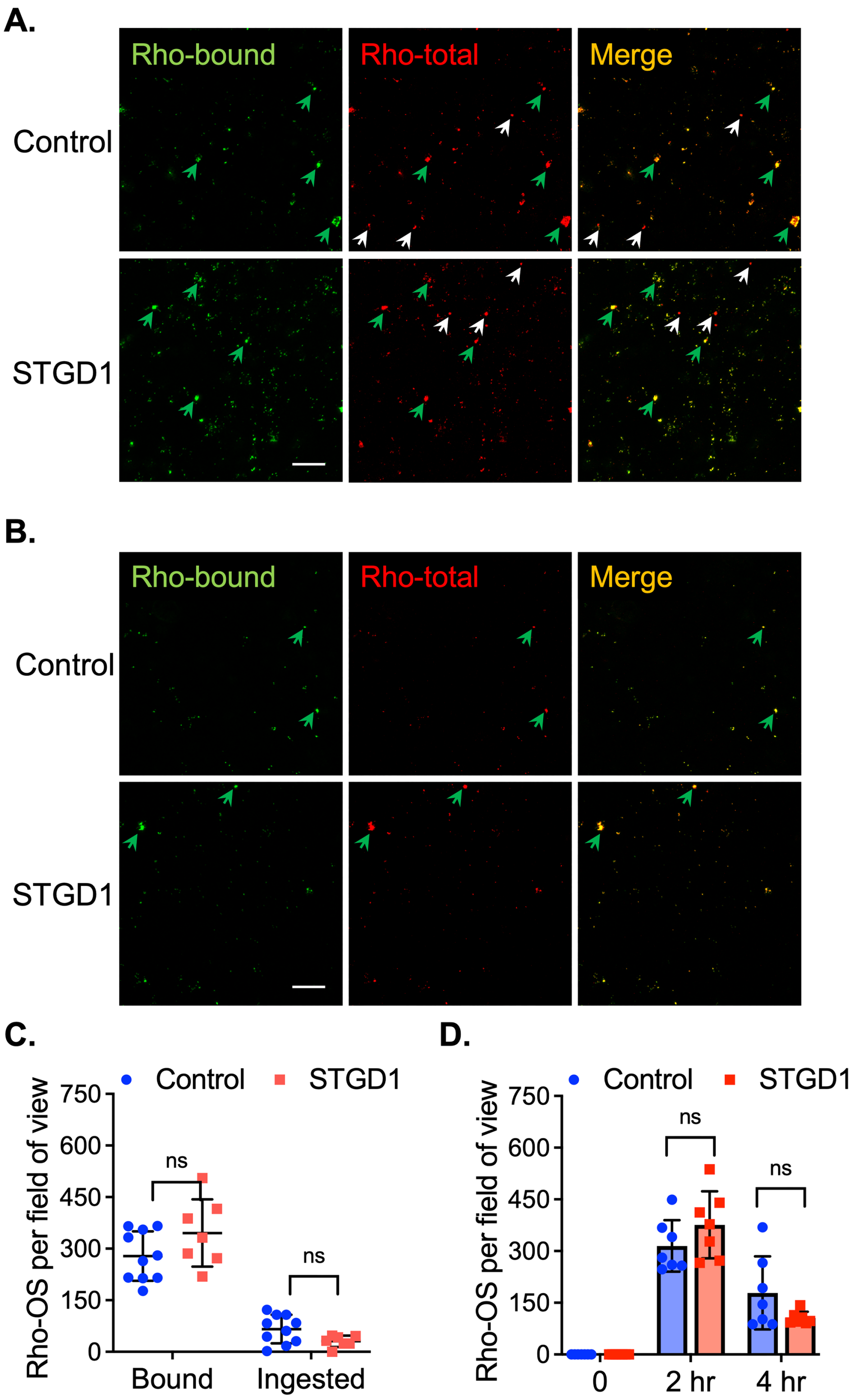

Supplementary Figure S2. Pulse-chase phagocytosis assay for human RPE cells.

Representative confocal microscopy images of control (top row) and STGD1 (bottom row) RPE

cells following incubation with bovine OS for two hours (2hr) pulse (**A**), then chased for additional two hours (**B**) after removal of unincorporated OS (4hr, the total time from the initial start time at 0hr). Rhodopsin staining was done with a primary anti-mouse rhodopsin (Rho) antibody and two secondary antibodies: (*i*) before permeabilization: Alexa Fluor 488 nm-conjugated goat anti-mouse IgG (green) and (*ii*) after permeabilization: Alexa Fluor 568 nm-conjugated goat anti-mouse IgG (red) to allow quantification of the bound (green only, before permeabilization-left images) and total (red, after permeabilization-center images) OS respectively. When merging red-green signals (right images), surface-bound OS appear yellow (green arrows) while internalized OS particles appear red only (white arrows). Degradation of Rho at 4hr (B) appear to be completed as no red only staining particles were observed. Scale bar = 20 μm. The amount of bound Rho-containing OS (green channel) was determined before permeabilization of the cells, while the amount of ingested Rho-containing OS was represented by the difference between total Rho-containing OS (permeabilized cells, red channel) and bound Rho-containing OS (green channel). Histograms show levels of bound and ingested Rho-OS per field of view quantified after 2hr-pulse phase (**C**) and total Rho OS per field of view quantified at both 2hr-pulse and 4hr-chase phase respectively (**D**). Quantification of Rho-OS was performed using the ImageJ software to count Rho-positive particles with diameters greater than 0.5 μm. Phagosome counts were obtained from 5-10 individual fields of view for each group. Data expressed as mean ± SD; n=3 filter/transwell per genotype; ns = not significant. All RPE cells were grown in culture for two-months *without* any retinoid supplementation.

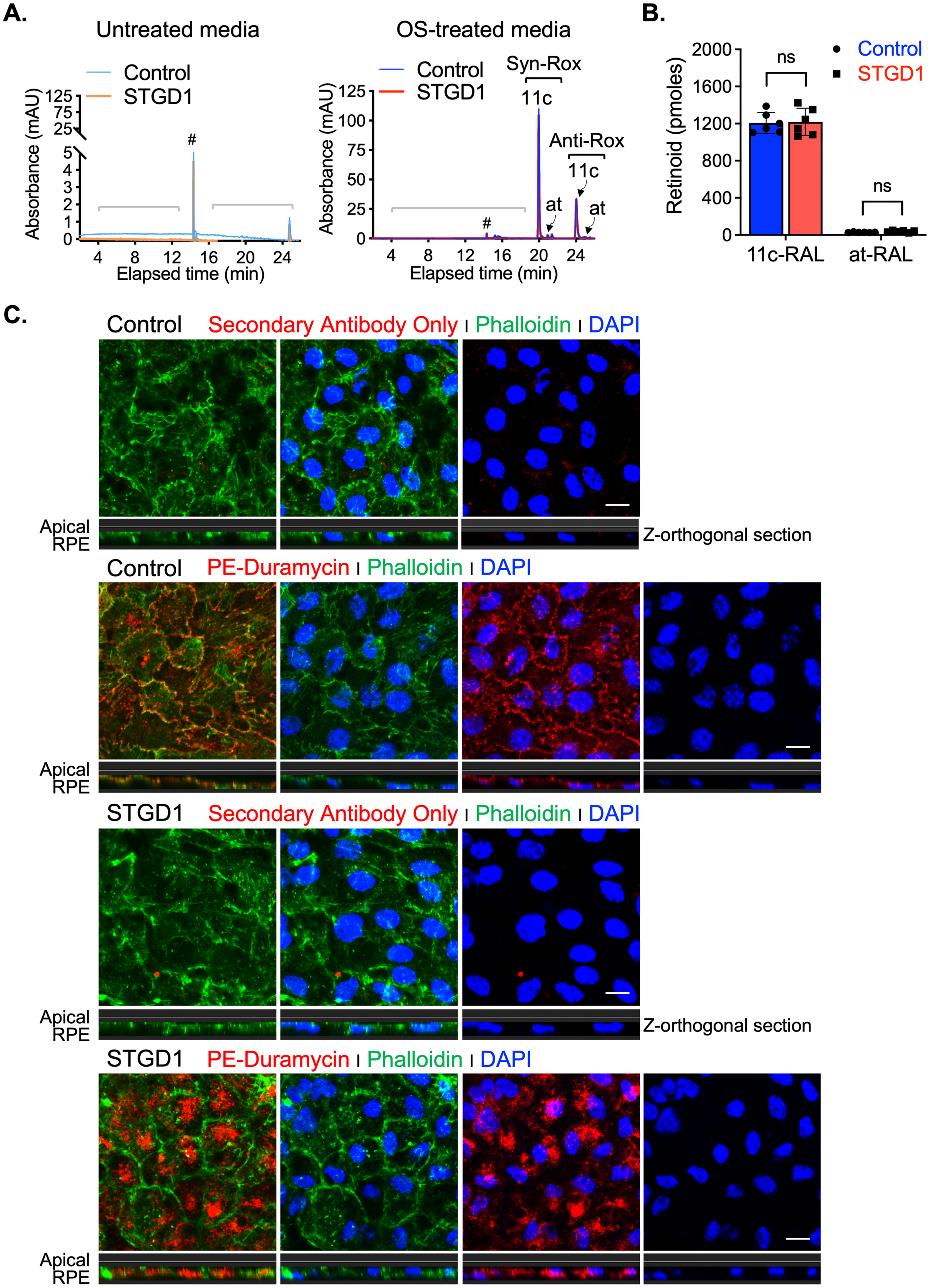

Supplementary Figure S3. ABCA4-flippase activity in the RPE cells. (**A**) Representative HPLC chromatograms at 350nm of hexane extracts of media from untreated (left) and OS-

treated (right) of three-months control (cyan/blue traces) and STGD1 (orange/red traces) RPE cells. Note that untreated cells (left) have *no* detectable retinoids in the media. Brackets indicate non-retinoid peaks based on spectral analysis and (#) indicates change in solvent gradient. Retinaldehyde were stabilized in the presence of hydroxylamine before the extraction in hexane and quantified as syn- and anti- retinaldehyde oximes (Rox). The peak height (absorbance in milli area units (mAU)) of syn- and anti- for both 11-*cis*- (11c-) and all-*trans*- (at-) retinaldehyde oximes (Rox) respectively were similar in the media of control (blue) and STGD1 (red) RPE cells. (**B**) Quantified levels of 11-*cis* (11c-) and all-*trans*- (at-) retinaldehyde (RAL) in the apical media were comparable in the control and STGD1 RPE cells. Note: RPE cells were grown in culture for three-months *without* any retinoid supplementation prior to this experiment. Data were expressed in pmoles as mean ± SD; n=6 filter/transwell per genotype; ns = not significant. (**C**) Representative confocal images of Secondary antibody only (red) and Phalloidin (green) for Control (1st row) and STGD1 (3rd row) RPE cells without the primary antibody to stain the PE-Duramycin. RPE cells were grown in culture for six-months in the presence of bovine retinal extract (~1.5 pmoles per feeding twice per week) supplemented at two-months. Individual channels of representative confocal images of PE-duramycin (red) and Phalloidin (green) staining corresponding to the merge image shown in main Fig. 2E. DAPI (blue) stains the nuclei. n=3 filter/transwell per genotype; Scale bar = 10 μm.

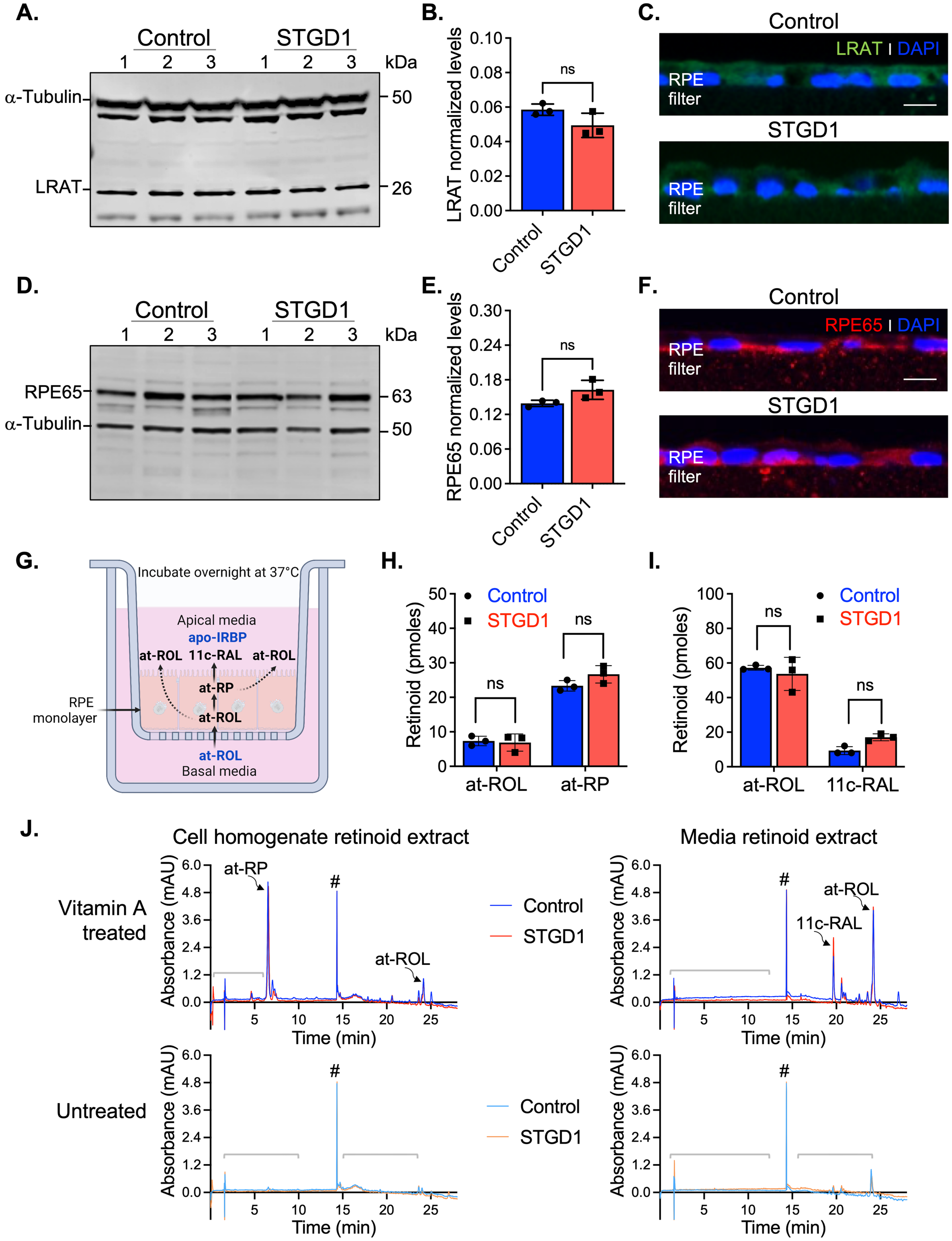

A.
Control
STGD1
1 2 3 1 2 3
kDa
α-Tubulin
LRAT
50
26
B.
LRAT normalized levels
ns
Control
STGD1
C.
Control
LRAT I DAPI
RPE
filter
STGD1
D.
RPE65
α-Tubulin
63
50
E.
RPE65 normalized levels
F.
RPE65 I DAPI
G.
Incubate overnight at 37°C
Apical media
apo-IRBP
at-ROL 11c-RAL at-ROL
at-RP
at-ROL
RPE monolayer
Basal media
H.
Retinoid (pmoles)
at-ROL
at-RP
I.
11c-RAL
J.
Cell homogenate retinoid extract
Media retinoid extract
Vitamin A treated
Untreated
Absorbance (mAU)
Time (min)
#

Supplementary Figure S4. Visual cycle proteins and activities in the RPE cells at three-months in culture. Representative immunoblots for LRAT/α-tubulin (**A**) and RPE65/α-tubulin (**D**) and corresponding normalized LRAT (**B**) and RPE65 (**E**) protein levels of control and STGD1 RPE cells homogenates. Data were expressed as mean ± SD; n=3 filter/transwell (corresponding to numbers one to three independent transwell on the blot) per genotype; ns = not significant. Representative confocal microscopy images of RPE cells for control (top) and STGD1 (bottom) sections immunostained for LRAT (**C**) and RPE65 (**F**). Scale bar = 20 μm. (**G**) Schematic diagram showing the experimental conditions to evaluate vitamin A uptake and processing via visual cycle for control and STGD RPE cells. All-*trans*-retinol (at-ROL, 10 μM), was delivered in the basal media (DMEM) supplemented with 1% BSA (fatty acid free) while apo-IRBP (10 μM) was added to the apical media to protect the released retinoids, at-ROL and 11c-RAL, by the RPE cells during overnight incubation at 37^0C. Retinoids were extracted in hexane from RPE cell homogenates (**H**) and corresponding media (**I**) and analyzed by normal-phase HPLC. Diagram created with BioRender.com. (**H**) Levels of retinoids in the RPE cells homogenates of control and STGD1 were similar for the at-ROL and all-*trans*-retinyl palmitate (at-RP). (**I**) Levels of the retinoids released in the media, at-ROL and 11-*cis*-retinaldehyde (11c-RAL), were also similar for the control and STGD1 RPE cells. (**J**) Representative HPLC chromatograms of hexane extracts of three-months cell homogenate at 325nm (left) and apical media at 350nm (right) of untreated (bottom) and vitamin A-treated (top) Control and STGD1 RPE cells respectively. No retinoids are detected in the untreated cells (bottom left) and corresponding media (bottom right) in either Control (light-blue trace) or STGD1 (orange trace) RPE cells. Brackets indicate non-retinoid peaks based on spectral analysis and (#) indicates change in solvent gradient. Note: RPE cells were grown in culture for three-months *without* any retinoid supplementation prior to this experiment. Data were expressed in pmoles as mean ± SD; n=3 filter/transwell per genotype; ns = not significant.

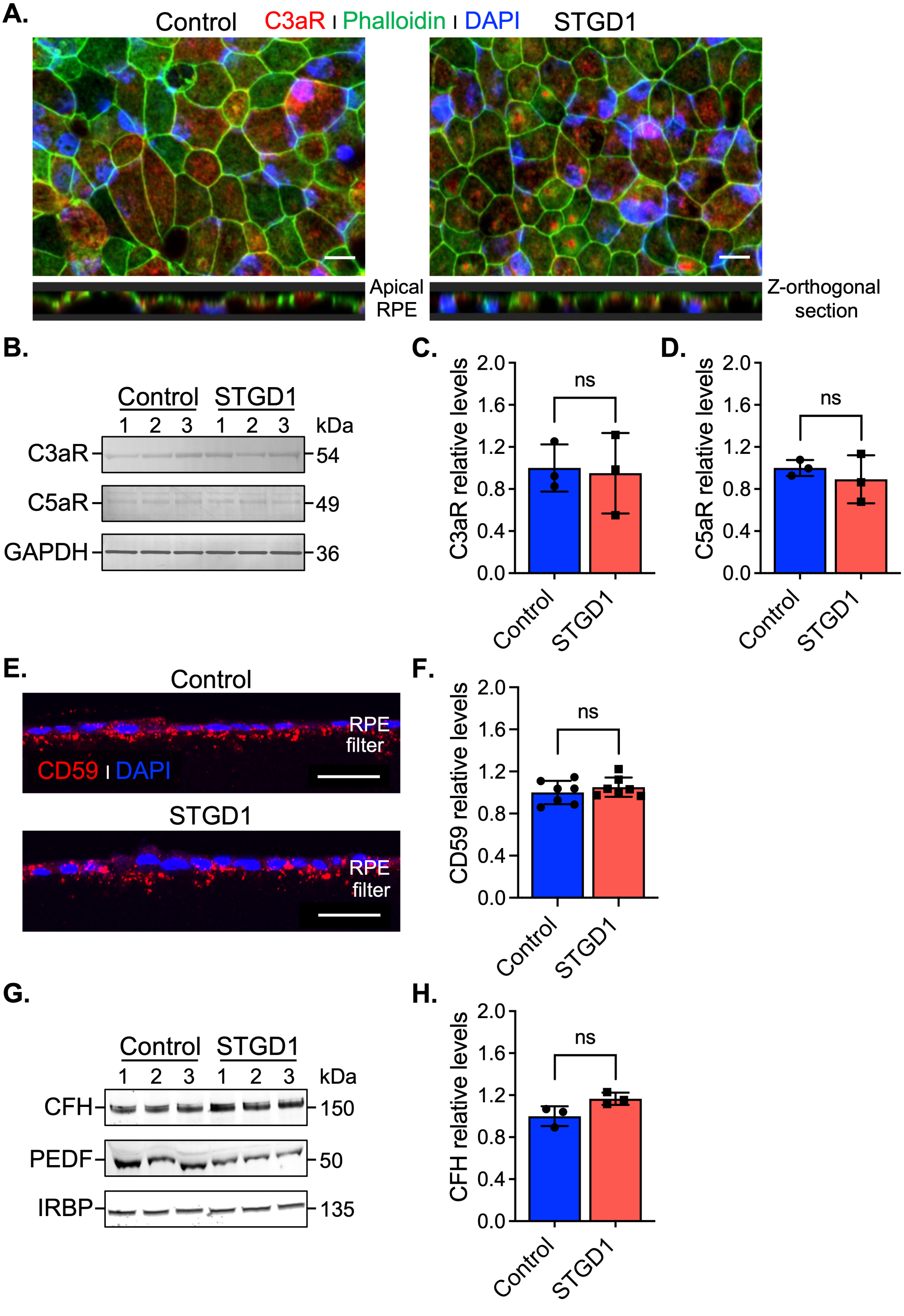

A.
Control
C3aR | Phalloidin | DAPI
STGD1
Apical RPE
Z-orthogonal section
B.
Control
STGD1
1 2 3 1 2 3
kDa
C3aR
54
C5aR
49
GAPDH
36
C.
C3aR relative levels
ns
Control
STGD1
D.
C5aR relative levels
ns
Control
STGD1
E.
Control
RPE filter
CD59 | DAPI
STGD1
RPE filter
F.
CD59 relative levels
ns
Control
STGD1
G.
Control
STGD1
1 2 3 1 2 3
kDa
CFH
150
PEDF
50
IRBP
135
H.
CFH relative levels
ns
Control
STGD1

Supplementary Figure S5. Complement C3a/C5a, CD59, and secreted proteins profile in human RPE cells. (**A**) Representative confocal images of control (left) and STGD1 (right) RPE cells immunostained for C3aR (red) and phalloidin (green). Nuclei were stained with DAPI (blue). Scale bar = 10 μm;. (**B**) Representative immunoblots of homogenates of RPE cells with C3aR, C5aR, and GAPDH. Histograms on the right shows the relative levels of C3aR (**C**) and C5aR (**D**) to control cells respectively after normalization to internal marker GAPDH. Average data shown as mean ± SD; n=3 filter/transwell (corresponding to numbers one to three on each blot) per genotype. (**E**) Representative confocal images of control (top) and STGD1 (bottom) RPE cells immunostained for CD59 (red), an inhibitor of the last step of the terminal complement complex. Nuclei were stained with DAPI (blue). Scale bar = 20 μm. (**F**) Histogram shows the relative levels of CD59 based on pixel intensity. Average data presented as mean ± SD; n=3 filter/transwell per genotype. (**G**) Representative immunoblots of total media (combined apical and basal) of RPE cells with antibody against major secreted proteins: complement factor H (CFH) and pigment epithelium derived factor (PEDF), with inter-photoreceptor binding protein (IRBP) as internal control. Each lane was loaded with 40 μg total protein of media sample. Numbers one to three on each blot represent independent biological samples for each genotype. (**H**) Relative levels of CFH are shown in the histogram after normalization to internal marker IRBP. Average data presented as mean ± SD; n=3 filter/transwell per genotype. ns = not significant. All RPE cells were grown in culture for three-months in the presence of bovine retinal extract (~1.5 pmoles per feeding, twice per week) supplemented at two-months. Note: taken together these findings suggest that C3a/C3aR and C5a/C5aR signaling pathways are not active players to STGD1 pathology. Further, support for CD46 complement dysregulation in STGD1 is signified as levels of CD59 and CFH, both negative complement regulators, are similar in control and STGD1 RPE cells.

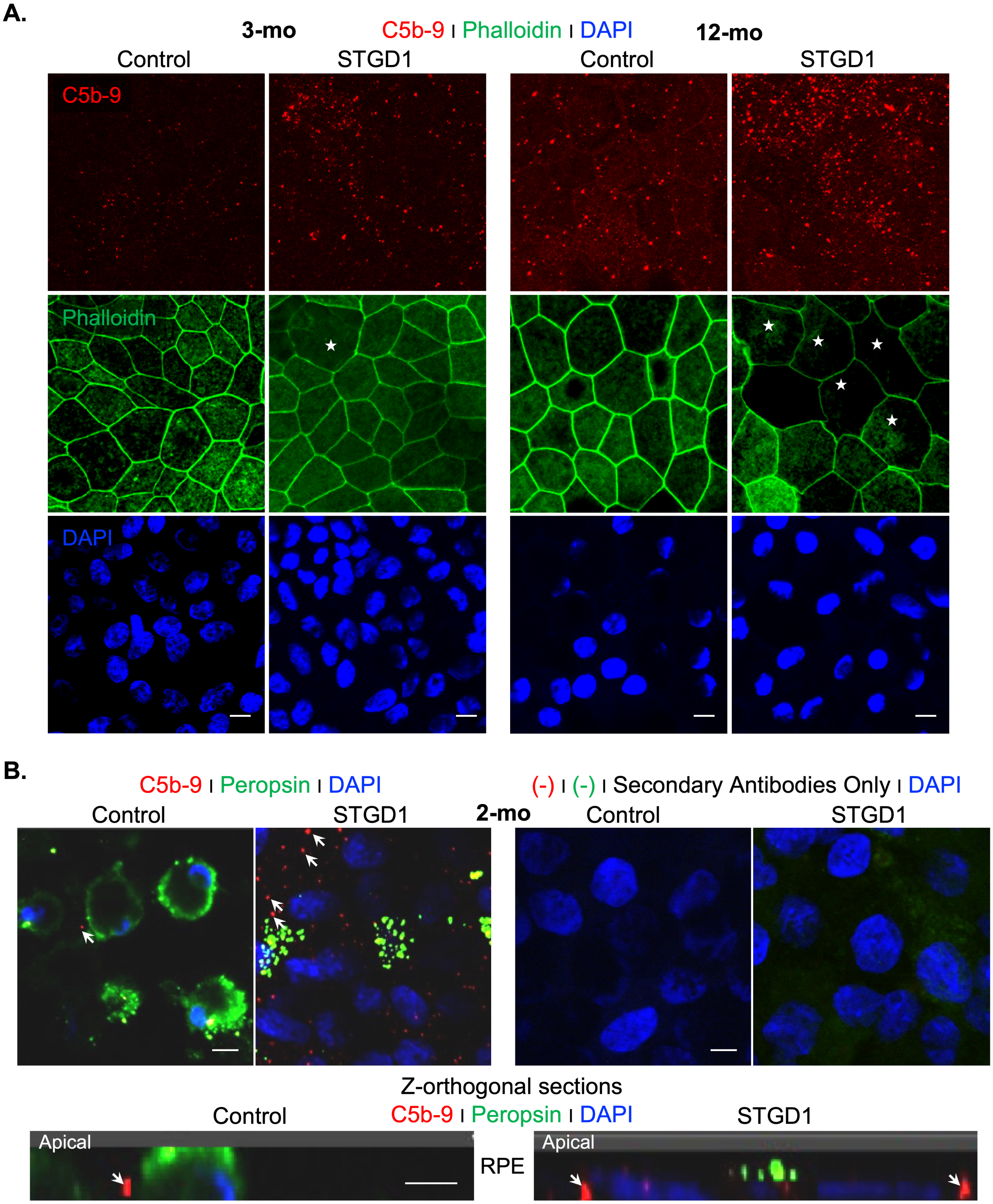

Supplementary Figure S6. MAC (C5b-9) deposition accumulates in STGD1 RPE cells.

(**A**) Representative en-face confocal images of individual channels for C5b-9 (red, top row), Phalloidin (green, middle row), and DAPI (blue, bottom row) acquired from control (left) and

STGD1 (right) corresponding to the merge images shown in main Fig. 6A. RPE cells were grown in culture for three-months (3-mo, on the left) and 12-months (12-mo, on the right) in the presence of bovine retinal extract (~1.5 pmoles of retinoids per feeding, twice per week) supplemented at two-months. En-face images of 12-mo STGD1 RPE cells displayed numerous larger cells (white stars) delineated by the Phalloidin staining. Scale bar = 10 μm (**B**) Representative confocal merge en-face images of C5b-9 (red) and Peropsin (green) from control (left) and STGD1 (right) RPE cells in culture for two-months. Corresponding Z-orthogonal sections are shown below the en-face confocal images. Secondary antibodies only representative en-face images are shown on the right (no primary antibodies). Note the abundancy of C5b-9 immunostaining (red) both in the en-face (A and B) and Z-orthogonal section (B) of the STGD1 RPE cells. In respect to peropsin staining, a protein that specifically localized only in the apically microvilli of the RPE cells, the MAC deposits appear predominantly intracellularly and basolaterally (indicated by the white arrows, in the Z-orthogonal sections). DAPI (blue) stains the nuclei. Scale bar = 10 μm; n=3 filter/transwell per genotype.

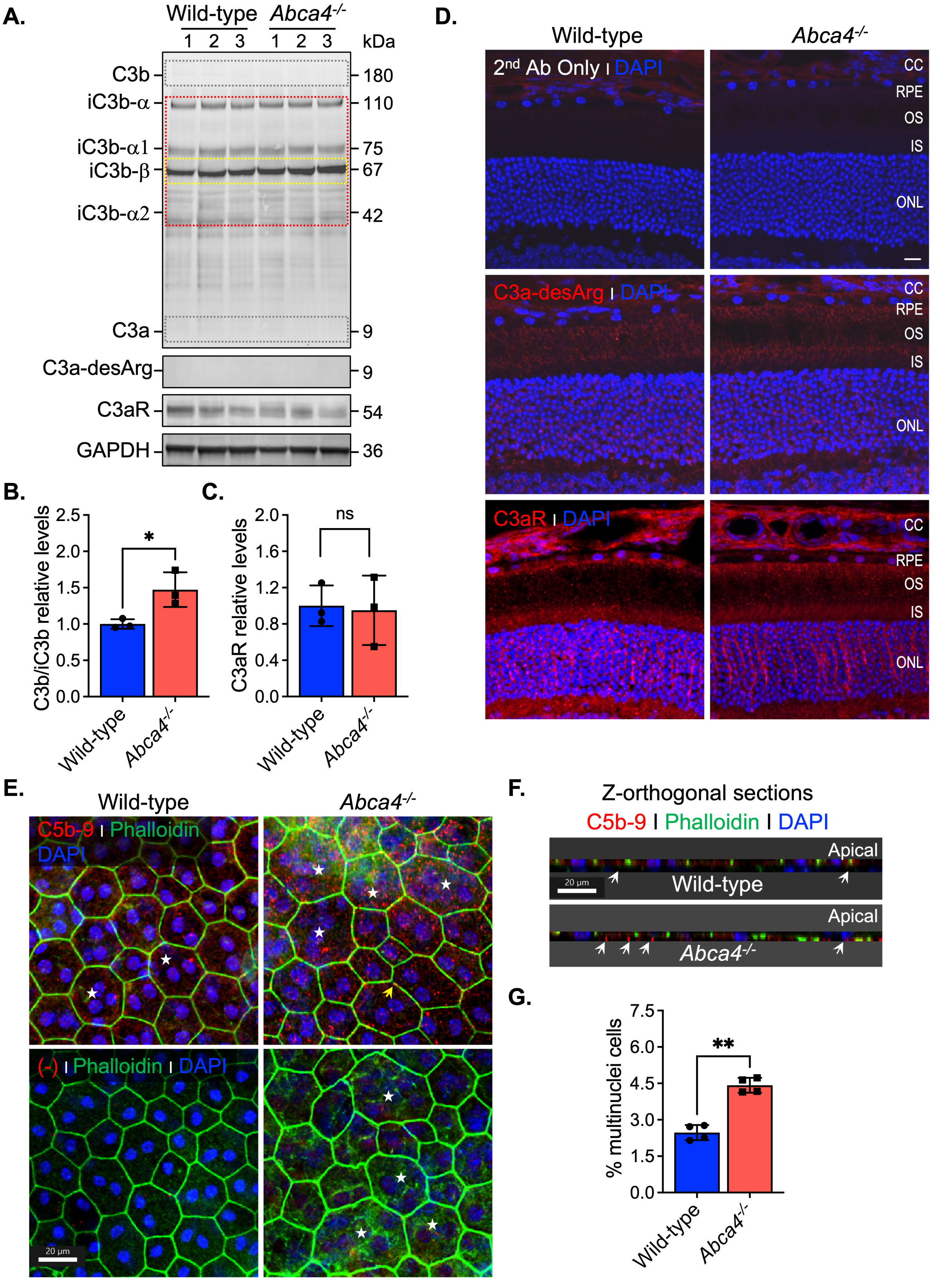

Supplementary Figure S7. Complement terminal complex destabilizes the RPE plasma

membrane of the *Abca4*$^{-/-}$ mice. (**A**) Representative immunoblots of RPE homogenates of six-months old albino wild-type and *Abca4*$^{-/-}$ mice using C3, C3a-desArg, C3aR, and GAPDH antibodies. Under reducing conditions, C3 antibody recognizes C3b, iC3b and C3a fragments. Histograms show quantification of the C3b fragment (**B**) and C3aR (**C**) respectively both normalized to GAPDH. Data presented as mean ± SD; **p*<0.05 (*p*=0.04); ns = not significant; n=3 mice per group. Numbers one to three on each blot represent independent biological samples for each genotype. (**D**) Representative confocal images of six-months old wild-type (left) and *Abca4*$^{-/-}$ (right) retina sections immunostained with an antibody against C3a fragment (C3a-desArg, 2nd row, red) and its cognate receptor C3aR (3rd row, red). DAPI (blue) stains the nuclei. Secondary antibodies control sections (without C3a-desArg and C3Ra primaries) are shown in the first row. Similar staining is observed in both genotypes. Scale bar = 10 μm. (**E**) Immunostaining of the terminal complement complex C5b-9 (or MAC, in red) and phalloidin (green) in RPE flatmounts of six-months old wild-type (left) and *Abca4*$^{-/-}$ (right) mice. Secondary antibody only (without C5b-9 primary antibody) representative confocal images are shown in the row below. DAPI (blue) stains the nuclei; Scale bar = 20 μm. Note numerous larger and multinucleated RPE cells (containing three or more nuclei) are evidenced in *Abca4*$^{-/-}$ flatmount indicated by the white stars. Also, intracellular C5b-9 accumulation (red only) and colocalization of C5b-9 and phalloidin (orange) is also more evidenced in the *Abca4*$^{-/-}$ flatmount. In the *Abca4*$^{-/}$, apparent breakage of RPE cells boundaries is indicated by the yellow arrow. (**F**) Corresponding Z-orthogonal sections of mouse RPE flatmounts show localization of C5b-9 (red, indicated by the white arrows) on the lateral site with a higher abundancy in the *Abca4*$^{-/-}$ vs wild-type. Scale bar = 20 μm. (**G**) Average data (mean ± SD) of multinucleated cells (containing three or more nuclei) quantification presented as percent of total RPE cells from a six to 20 images of each genotype; ***p*<0.005; n=4 mice per group.

References

1. Sun H, and Nathans J. Stargardt's ABCR is localized to the disc membrane of retinal rod outer segments. *Nat Genet.* 1997;17(1):15-6.
2. Li H, Tran VV, Hu Y, Mark Saltzman W, Barnstable CJ, and Tombran-Tink J. A PEDF N-terminal peptide protects the retina from ischemic injury when delivered in PLGA nanospheres. *Exp Eye Res.* 2006;83(4):824-33.
3. Sun H, Gilbert DJ, Copeland NG, Jenkins NA, and Nathans J. Peropsin, a novel visual pigment-like protein located in the apical microvilli of the retinal pigment epithelium. *Proc Natl Acad Sci U S A.* 1997;94(18):9893-8.
4. Mata NL, Moghrabi WN, Lee JS, Bui TV, Radu RA, Horwitz J, et al. Rpe65 is a retinyl ester binding protein that presents insoluble substrate to the isomerase in retinal pigment epithelial cells. *J Biol Chem.* 2004;279(1):635-43.
